# Bridging Global Frameworks and Local Practice: Quantitative Evaluation of Electronic Health Record Safety in Kuwait’s Public Hospitals

**DOI:** 10.2196/70782

**Published:** 2025-08-14

**Authors:** Anwar AlHussainan, Dari Alhuwail

**Affiliations:** 1 Information Science Department, College of Life Sciences, Kuwait University, Sabah AlSalem University City, P.O. Box 5969, Kuwait City, 13060, Kuwait, 965 24633096; 2 Health Informatics Unit, Dasman Diabetes Institute, Kuwait City, Kuwait

**Keywords:** electronic health records, safety assurance factors for electronic health record resilience, patient safety, risk assessment, electronic health record safety practices, public hospitals

## Abstract

**Background:**

Electronic health records (EHRs) play a critical role in today’s health care by enhancing data management, improving workflows, and supporting clinical decision-making. However, EHR implementation introduces technical and clinical challenges that can compromise patient safety. The Safety Assurance Factors for Electronic Health Record Resilience guides, developed by the Office of the National Coordinator for Health Information Technology, provide a structured framework for evaluating and optimizing EHR safety practices. Despite extensive research on EHR safety in developed countries, little is known about its implementation in regions with differing health care systems, such as Kuwait.

**Objective:**

This study aims to examine the EHR safety across hospitals in the State of Kuwait via (1) conducting a proactive risk assessment examining current safety practices and (2) proposing recommendations to improve EHR safety practices.

**Methods:**

A quantitative approach was used to evaluate EHR safety practices in 6 public hospitals. Multidisciplinary teams completed the Safety Assurance Factors for Electronic Health Record Resilience self-assessment questionnaire, scoring their implementation status of 165 recommended practices as “fully,” “partially,” or “not” implemented across 9 Safety Assurance Factors for Electronic Health Record Resilience guides. Data were analyzed to calculate the percentage of “fully implemented” recommended practices for each hospital, guide, and EHR safety domain. Standard deviations were calculated to assess data variability, and comparative analysis was conducted to identify implementation patterns.

**Results:**

The findings revealed significant variability in the implementation of recommended safety practices, with an average of 53% rated as “fully implemented” across hospitals. Infrastructure-focused guides, such as system configuration (77%) and system interfaces (80%), had the highest implementation rates, while clinical process guides, such as clinician communication (25%), scored the lowest. Among the 9 guides, 16 recommended practices were unanimously rated as “fully implemented,” while 8 were predominantly rated as “not implemented.” The high-priority guide showed notable variability, with implementation rates ranging from 17% to 89% across hospitals. Hospitals with longer EHR adoption periods tended to perform better, though hospital size and implementation type showed inconsistent effects on safety practices scores.

**Conclusions:**

The study highlights variability in EHR safety practice implementation across Kuwait’s public hospitals, with stronger performance in technical domains and gaps in clinical processes. By applying the Safety Assurance Factors for EHR Resilience guides in a non-US context, the study offers a foundational understanding of EHR safety implementation in Kuwait’s public health care system. Given the study’s limited scope and reliance on self-reported data, findings should be interpreted with caution. Future research should adopt broader sampling and mixed methods approaches to validate these results and inform the development of context-specific strategies to enhance EHR safety and patient outcomes.

## Introduction

Health IT is defined as “the application of information processing involving both computer hardware and software that deals with the storage, retrieval, sharing, and use of healthcare information, data, and knowledge for communication and decision making” to improve and maintain health care quality and patient safety [1,2]. Today, many hospitals rely on robust health IT solutions in their clinical practice, one of which is electronic health records (EHRs) [1,3].

In the United States, 78% of office-based physicians use EHRs in their medical practice [4-6]. EHRs include a set of documented information related to patient health care, such as demographics, progress notes, problems, medications, vital signs, medical history, immunizations, laboratory data, and imaging reports [7,8]. EHRs can also integrate with other advanced systems and features like clinical decision support (DS) systems, computerized physician order entry, health information exchange, and safety alerts [8,9].

With all these data, systems, and features, EHRs can support physicians in collecting medical history easily, providing better health care, and maintaining patient safety compared to relying on paper-based records [8]. Thus, EHRs contribute to improved data tracking and follow-up over time, which in turn helps guide health care providers (HCPs) in decision-making [8,10]. Moreover, the secondary utilization of EHRs offers the potential to transform existing data into actionable insights through health research, public health surveillance, quality improvement initiatives, and safety monitoring [11-13]. These applications are critical for identifying and addressing patient safety challenges within health care processes and for improving the quality of patient care [11,14].

The World Health Organization estimates that 1 in every 10 patients in developed countries is harmed while receiving health care in hospitals. Medical errors, often associated with diagnosis, prescription, and medication use [15], can be broadly categorized into two types: (1) adverse events that directly affect the patient, resulting in minor or severe injury or death, and (2) near misses that are intercepted before reaching the patient in unsafe conditions that elevate the risk of a future safety incident. These errors can lead to significant consequences, including fatalities, disabilities, poor health outcomes, increased health care costs, and legal complications [16-19].

A recent study in Kuwait found that approximately 60% of the frequency of medical errors ranged from incidences of prolonged hospital stays, adverse events, life-threatening complications, and fatalities. The causes of these medical errors included high workload, the lack of EHRs, HCPs’ non-compliance with safety guidelines, untrained or inexperienced administrative staff, and poor communication with patients and between HCPs [20]. While EHRs are designed to reduce the workload of HCPs and improve practice efficiency, cost, and error reduction, some studies reveal that implementing and using EHRs increases the work burden on HCPs [8,16,21,22].

In the United States, many physicians reported spending twice their working hours on EHR-related tasks, with some dedicating an additional 1-2 hours after work [8,23]. Consequently, this increased workload may elevate the risk of medical errors, ultimately compromising health care quality and patient safety [8,16,20,24]. Achieving the safe and effective use of EHRs presents several challenges, including usability issues, concerns about data integrity, safety risks, and the need for proper and ongoing system maintenance [10,25-29].

Evidence suggests that EHRs have demonstrated potential in reducing medication errors and adverse events and improving care quality [30-32]. In Kuwait, while health care professionals have shown acceptance of EHR adoption and expressed positive expectations, most hospitals remain in the early stages of implementation [33,34]. However, the adoption of EHRs introduces new risks that can adversely impact patient safety. These risks often stem from challenges related to system design, implementation, and usage. Specific issues include system downtime, poor usability, data entry errors, inadequate implementation processes, weak security measures, and malpractice by EHR users [35-37]. Addressing these risks is crucial to ensuring the safe and effective utilization of EHRs in health care settings [3,35].

Given that many health care organizations and HCPs prioritize patient safety, conducting regular assessments of EHR safety becomes essential [38]. EHR safety refers to the strategies and practices that ensure EHRs are used safely, securely, and effectively. These measures protect patients by preventing errors, harm, or any compromise to the quality of care [3,39-41]. The Office of the National Coordinator for Health Information Technology published a report highlighting key issues related to EHR safety and called for the development of proactive risk assessment tools [42].

One such tool is the Safety Assurance Factors for EHR Resilience (SAFER) guides, which provide a proactive risk evaluation framework that contributes to ensuring EHR safety, thereby supporting overall patient safety [42,43]. Furthermore, it is imperative to note that ensuring EHR safety requires a collaborative approach, with shared responsibilities between clinical and technical experts to optimize the use of EHRs [44].

In the State of Kuwait, the health care system is comprised of 2 main sectors: public and private. The public sector provides about 70% of health care services for citizens and residents, offering primary, secondary, and tertiary care within 6 health regions under the Ministry of Health [45,46]. Primary health care services are provided by polyclinics and health care centers located in residential areas. Notably, these centers and polyclinics offer initial diagnosis and treatment for patients, coordinating referrals to specialists in secondary and tertiary care if necessary. In contrast, secondary health care services are provided by 6 general hospitals distributed across the 6 governorates in Kuwait, while specialized health care services are offered by various specialized hospitals and centers [46-48].

This empirical study aims to conduct a proactive risk assessment examining the current EHR safety practices across public hospitals in Kuwait, with the goal of helping optimize EHR safety practices. To our knowledge, no prior studies have explored this topic in Kuwait or in comparable health care systems.

## Methods

### Ethical Considerations

This study was conducted in accordance with the Declaration of Helsinki, and the protocol was approved by the Standing Committee for Coordination of Health and Medical Research in the Ministry of Health in the State of Kuwait (1615/2021). All participants voluntarily signed the informed consent form before participating in the study procedures (Multimedia Appendix 1). Participants in the study were not paid for their participation. To maintain confidentiality and protect privacy, the names of the participating hospitals and health care staff have been anonymized throughout the study.

### Data Collection

This study used a quantitative approach to evaluate the current EHR safety practices among public hospitals in the State of Kuwait using the SAFER guides. [43,49,50]. Hospitals were selected based on the presence of EHR systems, whether fully or partially implemented. Initially, 6 public secondary care hospitals were targeted; however, 1 was excluded due to the absence of an EHR system. To maintain the sample size and ensure data relevance, a tertiary care hospital from the same health region was included as a substitute. The participating hospitals were targeted to complete the SAFER self-assessment questionnaire, rating their implementation status as “fully,” “partially,” or “not” implemented (Multimedia Appendix 2).

Each participating hospital was required to assemble a multidisciplinary team of health care staff, following a similar approach to a previous study [49]. A letter was sent to the hospital administration requesting the formation of a multidisciplinary team to complete the questionnaire or to designate an authorized and experienced individual who could assemble an appropriate team to evaluate the recommended practices using the SAFER guides, ensuring a comprehensive approach to patient care. This team typically included physicians, nurses, allied health professionals, and other specialists relevant to the study objectives.

Prior to data collection, the researchers conducted training sessions on the SAFER guides for the hospital teams, providing guidance and support to facilitate the completion of the questionnaires. During these sessions, a PowerPoint presentation was used to explain the purpose of the SAFER guides and the mechanism for completing the assessment. Additionally, researchers provided their contact information for assistance and distributed QR codes linking to instructional videos, the official SAFER guides website, a demographic information collection form for participating hospitals, and an Excel spreadsheet template of the SAFER questionnaire for data entry to ensure ease of use and consistency [49].

The SAFER guides, introduced in 2014 by the Office of the National Coordinator for Health Information Technology, were designed to support health care organizations in conducting proactive risk assessments of their EHR practices, with the goal of enhancing and advancing EHR safety. The SAFER guides include 165 recommended practices related to EHR safety, distributed across 9 guides [50]. Of these, 8 guides comprise 147 unique recommended practices, while the ninth, the high priority guide, consolidates 18 of the most critical recommended practices from the other guides.

In previous studies [49,51], the total number of recommendations was reported as 140 because the high priority guide was excluded from the count. Additionally, an audit and comparison of the official SAFER guides website with prior studies concluded that 7 recommendations were omitted due to thematic similarity with those already included in the high priority guide, which did not significantly impact SAFER guide scores. However, this study considers all 165 recommendations [50] to provide a more comprehensive assessment of EHR safety practices.

Overall, the SAFER guides are organized into three primary areas: (1) foundational guides, (2) infrastructure guides, and (3) clinical process guides. Foundational guides focus on high-priority practices and organizational responsibilities. Infrastructure guides involve contingency planning, system configuration, and system interfaces. Clinical process guides focus on areas such as patient identification, computerized provider order entry (CPOE) with DS, test results reporting and follow-up, and clinician communication. Each guide includes between 12 and 29 recommended practices, structured into 3 domains: Safe Health IT, Using Health IT Safely, and Using Health IT to Monitor and Improve Safety [42,43,49,52].

### Data Analysis

Following a similar analysis conducted in a previous study [49], the collected data were analyzed as follows: (1) calculated the percentage of “fully implemented” recommended practices across all 9 guides for each hospital, along with the standard deviation to measure data dispersion; (2) determined the mean percentage of “fully implemented” recommended practices across all participating hospitals for each guide; (3) assessed the percentage of “fully implemented” recommended practices across all participating hospitals for each EHR safety-related domain; and (4) evaluated the percentage of “partially implemented” and “not implemented” recommended practices in the high priority guide for each participating hospital.

## Results

### Demographic Attributes of Participating Hospitals

In total, 6 public hospitals participated in the study by completing the SAFER self-assessment questionnaire: 5 hospitals providing secondary health care services and 1 hospital providing specialized tertiary health care services. The participating hospitals had varied characteristics with respect to size, EHR adoption experience, and EHR implementation type (hospital demographic information is shown in Table 1).

**Table 1.** Demographics of participating hospitals.

Participated hospitals (N=6)	Numbers of hospitals
Hospital type	
Secondary health care	5
Tertiary health care	1
Hospital size	
Small	1
Medium	3
Large	2
EHR adoption year	
2006	1
2008	1
2015	2
2018	1
2019	1
EHR implementation type	
Outsourced developed	5
In-house developed	1

Hospitals were classified by size based on the number of beds and the total number of health care staff, including clinical, technical, and administrative staff. Small hospitals had less than 200 beds and fewer than 3000 employees (n=1), medium hospitals had 200-799 beds and 3000-6500 employees (n=3), while large hospitals had 800 or more beds and over 6500 employees (n=2) [45].

The participating hospitals also differed in their EHR adoption experience, ranging from 3 to 16 years, with initial implementations occurring between 2006 and 2019. Their approaches to EHR implementation varied, with 5 hospitals outsourcing the process through contracts with private companies for system deployment and support. Of these, 3 hospitals used the same vendor, while the other 2 engaged different vendors. In contrast, 1 hospital implemented its EHR system in-house, relying on local support from Ministry of Health developers.

A total of 38 health care staff members, forming multidisciplinary teams, participated in completing the SAFER guides questionnaire. Of the participants, 20 were female and 18 were male. While 2 hospitals assembled multidisciplinary teams of 6 members each, the remaining 4 hospitals formed teams of 7 members each (participant demographic information is shown in Table 2).

**Table 2.** Demographics of health care staff participants across the 6 hospitals.

Health care staff (N=38)	H1 (n=7)	H2 (n=7)	H3 (n=6)	H4 (n=6)	H5 (n=6)	H6 (n=6)
Age group						
24‐34	—^a^	2	—	1	2	4
35‐45	4	5	3	4	3	2
46‐56	3	—	2	1	1	—
57‐67	—	—	1	—	—	—
Health care staff sections						
Information systems	1	3	1	1	1	1
Medical records andstatisticsc	1	1	1	1	2	2
Pharmacy	1	1	1	—	1	1
Nursing	2	—	—	—	—	—
Physicians	2	1	1	2	1	2
Laboratory	—	1	1	1	1	—
Radiology	—	—	1	1	—	—
Years of experience						
1‐11	1	5	1	1	2	4
12‐23	5	2	2	5	3	2
24‐35	1	—	3	—	1	—
Gender						
Female (n=20)	4	4	3	3	3	3
Male (n=18)	3	3	3	3	3	3

a Not applicable.

### SAFER Guide’s Responses

**Figure 1.**
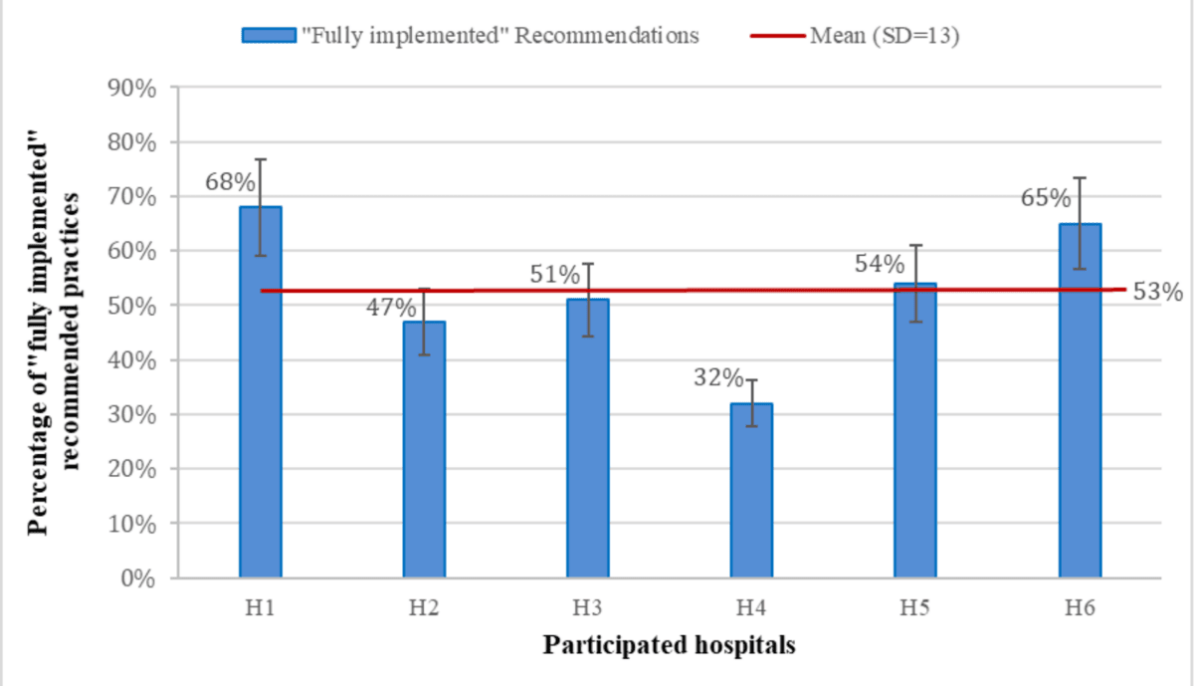
Percentage of “fully implemented” recommended practices across the 9 SAFER (Safety Assurance Factors for Electronic Health Record Resilience) guides for each hospital.

The percentage of “fully implemented” recommended practices across the 9 guides for each hospital, with a mean of 53% (SD 13), is shown in Figure 1. The highest percentage was 68% achieved by H1, while the lowest percentage was 32% achieved by H4.

**Figure 2.**
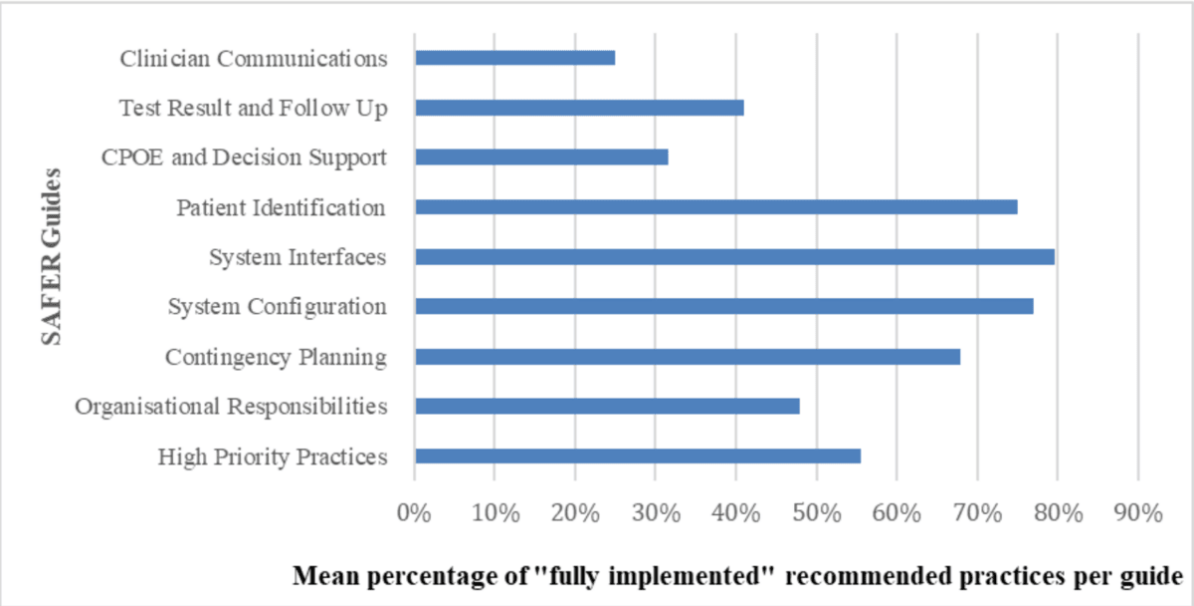
Mean percentage of “fully implemented” recommended practices of SAFER (Safety Assurance Factors for Electronic Health Record Resilience) guides across the 6 hospitals.

The mean percentage of “fully implemented” recommended practices of SAFER guides across the 6 hospitals is shown in Figure 2. The system interfaces guide, with 18 recommended practices, achieved 80% and ranked the highest, while the clinician communication guide, with 12 recommended practices, reached 25% and ranked the lowest. Among the 3 SAFER guides broad classifications, infrastructure guides (contingency planning [68%], system configuration [77%], and system interfaces [80%]) achieved the highest mean percentage of “fully implemented” recommended practices. Hence, foundational guides ranked second with an average of 52%, while clinical process guides ranked third with an average of 43%.

Furthermore, the safe Health IT domain, which comprised 54 recommended practices, achieved 66% of “fully implemented” recommended practices across the 6 hospitals, exceeding the Using Health IT to Monitor and Improve Safety domain, which comprised 23 recommended practices (54%), and Using Health IT Safely domain, which included 88 recommended practices (45%).

Regarding high-priority guide recommended practices, the percentage of implementation status for each hospital is shown in Table 3. The percentage of “fully implemented” recommended practices ranged from 17 to 89%, with H6 achieving the highest and H2 the lowest. While the percentage of “partially implemented” recommended practices ranged from 11 to 83%, only 2 hospitals had recommended practices rated as “not implemented”: H3 (22%) and H5 (11%).

**Table 3.** Percentage of implementation status for the high priority guide recommended practices for each hospital.

Implementation status	H1	H2	H3	H4	H5	H6
“Fully implemented”	78%	17%	67%	39%	44%	89%
“Partially implemented”	22%	83%	11%	61%	44%	11%
“Not implemented”	0%	0%	22%	0%	11%	0%

Examining hospital responses revealed that 16 recommended practices were unanimously rated as “fully implemented.” These recommended practices were distributed across 6 SAFER guides: high priority; contingency planning; system configuration; system interfaces; patient identification; and test results and follow-up. The system interfaces guide contained the highest number of recommended practices (n=5). On the other hand, 8 recommended practices were predominantly rated as “not implemented” by most hospitals. These “not implemented” recommended practices were drawn from 3 SAFER guides: CPOE/DS; test results and follow-up; and clinical communications, with the CPOE/DS and test results and follow-up guides each containing the highest number (3 recommended practices). Both “fully implemented” and “not implemented” recommended practices are provided in Tables4  5.

**Table 4.** SAFER (Safety Assurance Factors for EHR^a^ Resilience) guide recommended practices were rated “fully implemented” by all 6 hospitals.

SAFER guide	SAFER recommendation
Total of 16 “fully implemented” recommended practices	
High priority	“Information required to accurately identify the patient is clearly displayed on screens and printouts.”
Contingency planning	“Paper forms are available to replace key EHR functions during downtimes.” “Patient data and software application configurations critical to the organization’s operations are backed up.” “Policies and procedures are in place to ensure accurate patient identification when preparing for, during, and after downtimes.”
System configuration	“There are an adequate number of EHR access points in all clinical areas.” “The EHR is hosted safely in a physically and electronically secure manner.” “System hardware and software required to run the EHR (eg, operating system) and their modifications are tested individually and as-installed before go-live and are closely monitored after go-live.”
System interfaces	"System-to-system interfaces are properly configured and tested to ensure that both coded and free-text data elements are transmitted without loss of or changes to information content.” “Changes to hardware or software on either side of the interface are tested before and monitored after go-live.” “The operational status of the system interface is clear to its users with regard to clinical use, such as knowing when the interface cannot transmit or receive messages, alerts, or crucial information.” "The organization monitors the performance and use of system interfaces regularly, including monitoring the interface error log and the volume of transactions over the interface.” “When interface errors are detected, they are reported, fixed, and used to construct new test cases to improve the interface testing.”
Patient identification	“Patient names on adjacent lines in the EHR display are visually distinct.” “Medical record numbers incorporate a “check digit” to help prevent data entry errors.” “Patients are registered using a centralized, common database using standardized procedures.”
Test result and follow-up	“After system changes in components or applications related to CPOE and diagnostic services, the data and data presentation are reviewed to ensure accuracy and completeness.”

a EHR: electronic health record.

**Table 5.** SAFER (Safety Assurance Factors for EHR^a^ Resilience) guides recommended practices were most likely rated “not implemented” at the 6 hospitals.

SAFER guide	SAFER recommendation
Total of 8 “not implemented” recommended practices	
CPOE and DS^b^	“Clinicians are required to re-enter their password, or a unique PIN, to “sign” (authenticate) an order.” “Users can access authoritative clinical reference materials directly from the EHR, including organization-specific information when available.” “Additional safeguards, such as double check by a second specialist, are implemented in the EHR before high-risk medications are prescribed.”
Test result and follow-up	"There is an EHR-based process for clinicians to either assign surrogates for reviewing notifications or enable surrogates to look at the principal clinicians’ inboxes.” “There are mechanisms to forward results and results notifications from one clinician to another.” "The EHR has the capability for the clinician to set reminders for themselves and other responsible clinical staff for future tasks to facilitate test result follow-up.”
Clinician communications	”Electronic message systems include the capability to indicate the urgency of messages.” “The EHR displays time-sensitive and time-critical information more prominently than less urgent information.”

a EHR: electronic health record.

b CPOE and DS: computerized provider order entry with decision support.

## Discussion

### Principal Findings

EHR implementation provides a valuable opportunity to optimize health care safety, services, procedures, processes, and outcomes [53]. The conducted assessment helps health care staff engage appropriately across various specialties and tasks to deliver better EHR safety, considering all relevant aspects. Data from the 6 hospitals were analyzed to evaluate the extent to which they have implemented the best recommended safety practices related to EHR adoption and usage, using the SAFER guides.

### SAFER Scores Variability

The percentage of “fully implemented” recommended practices from the SAFER guides across participating hospitals showed significant variability, with an average implementation rate of 53%, as illustrated in Figure 1. For instance, the implementation rates ranged from 4% to 92% for the organizational responsibilities guide, 23% to 100% for the system configuration, and 0% to 58% for the clinician communication guide. While previous studies mention that hospital size, years of EHR adoption, and type of EHR implementation affect EHR safety [54,55], the findings indicate an imperfect relationship between these factors (hospital demographics; Table 1) and the scores of “fully implemented” SAFER-recommended practices, with no clear and consistent direct or inverse relationship.

Hospitals with longer EHR adoption periods generally perform better, but variations in safety outcomes suggest that experience alone does not fully determine EHR safety. Similarly, in this study, hospital size showed no clear correlation with SAFER scores, as medium and large hospitals exhibit differing results likely influenced by other factors. The type of EHR implementation (in-house or outsourced) also did not directly predict SAFER scores, as both types displayed wide variations in implementation levels.

Accordingly, this variability highlights the significance of hospital-specific factors in determining EHR safety effectiveness. More influential than demographic characteristics alone, these factors include available resources (human and financial), safety culture, technical capabilities, workflow integration, quality of staff training, implementation strategies, and leadership commitment. These critical elements significantly impact EHR safety outcomes and the extent to which EHR safety measures and recommended practices are implemented [54,56,57].

### Infrastructure and Technical Focus

Out of the 165 recommended practices in the SAFER guides, only 16 (10%) were “fully implemented” by the participating hospitals. The researchers observed that all participating hospitals focus more on infrastructure and technical aspects across guides or domains. The implementation rate for infrastructure guides gets a higher mean percentage (75%), specifically system configuration (77%) and system interfaces (80%). In contrast, the clinical process guides had a much lower implementation rate of only 43%.

Additionally, the percentage of implemented recommended practices varied considerably across each different EHR-related patient safety domain. Domain 2—Using Health IT Safely, which focuses on ensuring the effective and safe utilization of EHRs by health care staff and patients—had an implementation rate of only 45%. Meanwhile, Domain 1—Safe Health IT, which emphasizes the technical protection aspects of EHR structure and functionality, reached 66%. Hospitals tend to be technically oriented for several reasons. Technical aspects, such as systems configuration, interface management, and interoperability, are fundamental to EHR functionality [58-60]. A secure, reliable, and robust technical setup is essential for the health care system to prevent failures and errors that could disrupt clinical care. Studies have emphasized the importance of solid testing environments in determining safety and usability issues during the system implementation phases [61-63].

Furthermore, EHR vendors can influence the hospitals’ technical orientation. Many hospitals rely on external technology vendors to implement, develop, and support their EHR systems [64,65]. However, these vendors often lack a deep understanding of the clinical intricacies of specific hospitals [66]. Instead, they prioritize technical capabilities and compliance standards—such as security, databases, interoperability, and system function and updates—that are directly manageable and required for system credentials and regulatory compliance with governmental regulations [67-69].

Moreover, hospitals may find that technical issues (eg, data security, usability, and privacy) are easier to address than clinical process issues (eg, clinician communication and workflow integration), particularly in the early test cases [70,71]. Technical adjustments are generally more straightforward to standardize, implement, measure, and audit, whereas clinical workflows are inherently more complex [72,73].

Lastly, there is a common misconception that technical stability, including a well-functioning EHR, guarantees health care safety [27,68]. Many hospitals assume that if their EHR system is secure, interoperable, and compliant, it will inherently be safe for health care staff use and patients [23,61,74,75]. Thus, hospitals prioritize technical improvements to avoid system failures and mitigate risks. However, these efforts alone cannot address other critical issues such as health care staff acceptance issues, communication breakdowns, and workflow discrepancies, which often pose more significant risks to patient safety [76-79]. Therefore, achieving EHR safety requires a comprehensive approach that addresses both technical and clinical process factors [80].

These factors contribute to the hospital’s tendency toward technical over clinical processes for EHR safety, which can lead to neglecting the significant effects on clinical practices and patient care. Addressing both aspects of the health care system is necessary for a robust and effective safety setting [80,81].

### Clinician Communication Challenges

Communication between health care staff, particularly clinicians, is a fundamental aspect of clinical processes encompassing critical tasks and decisions. Communication breakdowns can directly affect patient safety, leading to medical errors and patient harm [50,82]. The implementation rate for the clinician communication guide’s recommended practices was the lowest across the hospitals, ranging from 0% to 58%. This guide often receives the lowest scores in EHR safety evaluations due to several factors. First, there is a lack of robust integration features for communication tools. For instance, disjointed messaging systems lead clinicians to rely on multiple platforms or external solutions, and fragmented data through EHR’s various sections make it difficult to reach, particularly during clinical discussion communications [83].

Additionally, usability challenges, such as poorly designed interfaces and alert fatigue, impact the efficiency of clinician communication. Interfaces may include many steps or unclear options, making it difficult for clinicians to follow. An overabundance of notifications or irrelevant alerts may lead to ignored or missed important communications [25,84]. Moreover, inconsistent communication and time constraints lead to workflow misalignment, creating barriers to facilitating clinical workflows. EHRs are mostly designed for individual duties, prioritizing their development (eg, data or order entry and individual decision-making) rather than promoting collaborative communications between health care team members. EHR communication tools can increase clinicians’ burden, conflicting with their time-sensitive workflow [85,86].

Furthermore, health care staff attitudes and knowledge gaps, such as a lack of awareness and resistance to change, contribute to communication challenges. Clinicians may not have adequate awareness of using EHRs due to insufficient onboarding or training in EHR communication features. Many clinicians find that the traditional methods (eg, phone calls and face-to-face discussions) are easier and more effective than using EHRs [85,87]. Lastly, interdepartmental and interdisciplinary communication processes face significant obstacles. The lack of communication tools that meet various requirements for multidisciplinary teams hinders effective communication, especially among nonphysician or specialist staff. Besides, data may not be easily accessible or shareable across systems or roles within the same hospital [87]. These challenges should be considered, addressed, and improved to integrate seamlessly and effectively into clinical workflows. Developing clinician communications within EHRs requires simplified interfaces, adequate integration of collaborative tools, practical training, and adjustable workflow regulations [85,87].

### Comparison With Prior Work

Previous studies have applied SAFER guides as a methodology. A study evaluating 8 health care organizations in the United States found that, on average, only 18% of the 140 unique safety recommendations were fully implemented [49]. Adherence was higher in the Safe Health IT domain (82.1%) compared to Using Health IT Safely (72.5%) and Monitoring Health IT (67%). This aligns with our study’s findings, where technical infrastructure showed higher adherence, emphasizing a common trend of prioritizing technical domains over clinical processes and communication.

On the other hand, another study introduced a self-assessment focused on the Computerized Provider Entry system, using 2 SAFER guides—the CPOE with DS guide and clinician communication guide—which were filled out by nursing students [43]. The study demonstrated that such a tool could effectively uncover usability and safety issues, particularly medication ordering and visual display issues. It explored nursing students’ experiences with EHR safety, highlighting the importance of education and training in promoting safe EHR use. This underscores the need for comprehensive training programs to enhance EHR safety practices, a factor that may contribute to variability in adherence observed in our study. While our study did not focus on CPOE systems, the emphasis on self-assessment tools aligns with our study approach of using SAFER guides to proactively identify and address EHR-related safety concerns.

Several factors may be considered for the observed differences between our study and previous studies. The stage of experience with the system and EHR adoption can influence the implementation of safety recommendations [23,88]. Likewise, organizational commitment to implementing safety practices and the diversity of cultural attitudes toward patient safety can lead to variability in adherence levels [89]. Additionally, health care system differences between the State of Kuwait and other countries, such as variations in health care priorities, infrastructure, and policies, can impact the implementation of SAFER-recommended practices [90]. Furthermore, resource availability can influence the capacity to effectively implement EHR safety practices, including differences in technical, human, and financial resources [91,92].

### Recommendations

EHR safety encompasses organizational, clinical, technical, and behavioral dimensions, all of which are essential and interdependent in ensuring health care quality and patient safety [56]. Achieving EHR safety requires shared responsibility among interdisciplinary health care teams, fostering collaboration to enhance safety practices and integrate clinical and technical workflows seamlessly [44,87,93]. Additionally, promoting awareness of EHR safety through comprehensive and ongoing training for health care staff can improve their proficiency and confidence in using EHR systems [63].

Hospitals must also commit to regularly conducting proactive risk assessments, such as those outlined in the SAFER guides, to identify potential vulnerabilities in EHR safety practices. These assessments allow for the implementation of preventive measures, mitigating risks before adverse events occur and improving health care outcomes and patient safety. Proactive assessments can further foster interdisciplinary collaboration among HCPs by bringing together diverse expertise, supported by strong leadership to establish organizational priorities, promote workflow adaptability, and actively involve EHR vendors in optimization initiatives [51,78,94].

EHR vendors should gain a comprehensive understanding of clinicians’ workflows and the broader operations of health care teams to design systems that integrate effectively into clinical processes [86]. Actively involving health care staff as EHR users is critical, as they can provide valuable feedback to identify areas for improvement, emphasizing the need for established channels for user input [95].

Although not widely available at the time the SAFER guides were first introduced, advanced technologies such as artificial intelligence (AI) and machine learning (ML) now offer significant potential to enhance EHR safety [96,97]. Early evidence suggests that these technologies can improve DS, streamline workflows, and reduce clinician burden, particularly through advanced data entry automation and error detection mechanisms [98]. However, their impact on EHR safety remains in the early stages of evaluation, and further research is required to comprehensively understand their effectiveness.

Integrating AI and ML into the SAFER guides could provide a proactive framework for assessing and mitigating risks associated with these technologies while optimizing their benefits for EHR safety. This integration would support health care organizations in identifying best practices for leveraging AI and ML to address safety challenges, such as improving data integrity, detecting potential errors, and enhancing user experience. Future studies should focus on the long-term impact of AI and ML in real-world clinical settings and provide evidence-based recommendations for their inclusion in SAFER guides, thereby advancing EHR safety and patient care.

### Study Strengths and Limitations

As with any research, this study has both strengths and limitations. First, it represents the first empirical investigation into EHR safety practices in public hospitals outside the United States using the SAFER guides. The lack of similar international studies restricts opportunities for benchmarking and cross-context comparisons, limiting the ability to generalize findings beyond Kuwait.

Second, although the study included only 6 public hospitals, it effectively captured nearly the entire population of secondary care facilities in Kuwait, providing a comprehensive snapshot of this health care segment. One secondary hospital was excluded due to the absence of an EHR system and was replaced with a tertiary care hospital from the same region. While this maintained the sample size and focus on EHR-active facilities, it may have introduced variability, as tertiary hospitals typically differ in patient complexity, resource availability, and EHR usage. Future studies should stratify by care level and include tertiary and private hospitals to enhance generalizability.

Third, a significant limitation of the SAFER guides is the reliance on self-assessment, which may not always yield objective evaluations of EHR safety practices. Because the tool depends on health care staff to assess their own systems and processes, the accuracy of the results is influenced by participants’ understanding, perceptions, and interpretations of the recommended practices. This self-reporting approach introduces potential biases, as participants may overestimate adherence to best practices or fail to identify existing deficiencies. Lastly, the differences in health care system regulations and policies between Kuwait and the United States may affect how SAFER scores are interpreted and applied.

Nonetheless, the SAFER guides offer a valuable framework for health care staff and hospitals to identify gaps, enhance EHR safety practices, improve workflow efficiencies, and achieve better health care outcomes. To address these limitations, future studies should consider incorporating complementary methodologies, such as external audits, qualitative interviews, and observational studies. These approaches can validate findings from SAFER self-assessments, provide more objective evaluations, and yield deeper insights into the practical challenges and successes of implementing the recommended practices across diverse health care contexts. Additionally, studies focusing on the long-term impact of SAFER-guided interventions could assess improvements in patient safety and organizational efficiency, contributing valuable knowledge to the global discourse on EHR safety and optimization.

### Conclusions

This study used the SAFER guides as a structured framework to conduct a proactive risk assessment of EHR safety practices in Kuwait’s public hospitals. The findings reveal considerable variability in the implementation of recommended safety practices, with stronger adherence observed in technical and infrastructure-related domains compared to clinical processes and clinician communication. These results underscore the importance of addressing both technical and clinical dimensions to achieve a balanced and effective approach to EHR safety.

The study contributes to the growing body of literature on EHR safety by demonstrating the applicability of the SAFER framework in a non-US context. While the SAFER guides proved useful in identifying gaps and strengths in current practices, the results do not establish causal relationships between hospital characteristics and safety outcomes. Instead, they offer a foundational understanding of EHR safety implementation in Kuwait’s public health care sector. The unique regulatory and operational characteristics of Kuwait’s health care system necessitate tailored strategies for effective implementation. Future research should build on these findings through broader sampling, mixed methods approaches, and longitudinal assessments to evaluate the impact of targeted interventions on EHR safety and patient outcomes.

## Supplementary material

10.2196/70782 Multimedia Appendix 1 Informed consent form used for the survey.

10.2196/70782 Multimedia Appendix 2 SAFER (Safety Assurance Factors for EHR [Electronic Health Record] Resilience) guides (Self-evaluation Questionnaire).

## Abbreviations

AI: artificial intelligence
CPOE: computerized provider order entry
DS: decision support
EHR: electronic health record
HCP: health care provider
ML: machine learning
SAFER: Safety Assurance Factors for EHR Resilience
