# Supplementary material for: Bridging Global Frameworks and Local Practice: Quantitative Evaluation of Electronic Health Record Safety in Kuwait’s Public Hospitals
Source: JMIR Med Inform. 2025 Aug 14;13:e70782. doi: 10.2196/70782 (PMC12501905; doi:10.2196/70782)
Supplement: Multimedia Appendix 1 [file medinform-v13-e70782-s001.pdf]

Informed Consent Form for Survey

Informed Consent Form
(SAFER Self-evaluation questionnaire)

Title of the Study:
Examining Electronic Health Record Safety Practices across Hospitals in the State of Kuwait: An Empirical Study

What is the purpose of the Study?
This study is part of a thesis in the Master of Science Degree in Computing Information Systems at the College of Life Sciences – Kuwait University. This study aims to: **(i)** conduct a proactive risk-assessment examining current EHR safety practices in the public hospitals in the state of Kuwait, **(ii)** uncover the challenges and opportunities for achieving better EHR safety, and **(iii)** Propose recommendations to assist hospitals in improving their EHR safety practices.

Why have I been invited to participate?
You are being invited to participate in the study for your professional role as a part of healthcare staff (clinicians, pharmacy, diagnostic services, clinical administration, safety officers, administrators, health IT support staff, EHR developers) in hospitals in the State of Kuwait. Your participation is voluntary, and you have the right to accept or refuse to be a participant in the study at any point. Your refusal to participate will not affect your employment status or health benefits.

What procedures will be performed on me?
You are invited as a member of the necessary team of healthcare staff (clinicians, pharmacy, diagnostic services, clinical administration, safety officers, administrators, health IT support staff, EHR developers) assembled by the hospital to participate voluntarily self-evaluation questionnaire. A self-evaluation questionnaire will be conducted using Safety Assurance Factors for EHR Resilience (SAFER) guides to score the EHR implementation status in the hospital that you are rolling in as: "fully implemented", "partially implemented", or "not implemented". The questionnaire will be distributed in English (hard/soft copy). The researchers will consult with each hospital and assist with completing the questionnaire. No identifiable information will be shared with anyone outside of this study protocol. You may also be invited to a shorter 5-minute follow-up online meeting (i.e., member check) to verify your answers and potentially for clarifications.

SAFER guides were released in 2014 to assist healthcare organizations in performing a proactive risk assessment for their EHR practices to help in improving and developing EHR safety. SAFER includes 140 recommendations related to EHRs safety embedded in nine guides (range 10-29 recommendations per guide) that are divided into three main areas: (i) foundational guides, (ii) infrastructure guides, and (iii) clinical process guides. Foundational guides involved high priority practices and organizational responsibilities. Infrastructure guides involved contingency planning, system configuration, and system infrastructure. Clinical process guides involved

patient identification, computerized provider order entry with decision support, test results reporting and follow-up, and clinician communication. The recommendations in each guide are organized into three domains: Safe Health IT (total 45 recommendations), Using Health IT Safely (total 80 recommendations), and Using Health IT to Monitor and Improve Safety (total 15 recommendations).

What are the benefits to me for taking part in this study?
By participating in this study, you will not gain any personal benefits. However, the findings from this research will help researchers conduct a proactive risk-assessment examining current EHR safety practices in hospitals in Kuwait.

What are the risks to me for taking part in this study?
By participating in this study, you will gain no harm but maybe feel discomfort due to the length of the self-evaluation questionnaire.

What will happen to the information I provide? (Confidentiality)
The entire pool of collected data, like questionnaires, interviews, consent forms, and any other related documents, will be stored in secured files and devices and identified only by a participant identification number. Private information will not be shared or revealed to a third party without the participants' written permission, and it will not be used in any scientific publication or presentations. When a participant wishes to discontinue the questionnaire, researchers will inform the participant that there will be no penalty. The participant has the right to request not to use the provided answers.

Who do I contact if I have concerns or want further information?
Researcher Name: Anwar AlHussainan– email: anwar.alhusainan@grad.ku.edu.kw
Supervisor's name: Dr. Dari Alhuwail – **Tel.:** 24633095 – **email:** dari.alhuwail@ku.edu.kw
By signing, I consent to participate in the above-mentioned study.
Date: …………………
Participant Name: ………………………… **Signature:** …………………
Researcher: ………………………………… **Signature:** …………………
